# The Coming Meeting; Bury the Hatchet

**Published:** 1888-04

**Authors:** 


### EDITORIAL DEPARTMENT.

F. E. DANIEL, M. D., EDITOR AND PUBLISHER

THIS JOURNAL, by unanimous vote of the members, was made the Official Organ of the Austin District Medical Society.

COLLABORATORS:

*Dr. R. M. Swearingen., Austin.*
*Dr. B. E. Hadra, Austin.*
*Dr. Geo. Cupples, San Antonio.*
*Dr. T. C. Osborn, Cleburne, Texas.*
*Dr. E. J. Doering, Chicago.*
*Dr. Odo Betz, Germany.*
*Dr. J. W. McLaughlin, Austin.*
*Dr. T. J. Tyner, Austin.*
*Dr. J. F. Y. Paine, Galveston.*
*Dr. E. Goldmann, San Jose, Cal.*
*Dr. H. O. Marcy, Boston.*
*Dr. E. Meierhof, New York.*
*E. J. Beall, M. D., Fort Worth, Texas.*

### THE COMING MEETING.—BURY THE HATCHET.

From a very extensive correspondence with members of the State Association, in all parts of the State, we are satisfied that our views are endorsed, as expressed in an article on the importance of preserving harmony in the ranks—and conducting the next meeting solely in the interest of medical science, to the exclusion, as far as may be, of personal grievances ; there is a very general expression of a desire in this direction, and all deprecate the occurrence of the disturbing influences introduced in our last.

It is particularly gratifying to cite, as one of many such endorsements, the following, from a prominent and popular physician, couched in language the eloquence and pathos of which must touch the heart of every lover of his profession. He says :

" I read with much interest and pleasure your leading article. It strikes the "key-note" of the occasion, and will doubtless be productive of much good. No words or sentences could have been assembled that would have so clearly lifted the mantle of ambiguity from the situation ; and, being supported by an opportune and eloquent appeal to the better element, and loftier spirit of our natures, leaves nothing to be said.

"For this appeal, which must touch a responsive chord in the hearts of all true and honorable physicians, you are entitled to the cordial thanks of all members of the Association, whose aspirations rise above the venom of personal and professional prejudice and jealousy.

"No greater calamity could befall the profession of a State, and through this channel, the scientific and practical interests of the people of that State, than for its recognized and influential State Medical Association—which has been led to the van of all similar institutions through the labors of a score of years—to virtually acknowledge its inability to preserve its integrity, and perpetuate its usefulness—to suffer a dagger, poisoned with the virus of personalities, unchaste and undignified aspersions, to penetrate its vitals, signalize extinction, and deflower its lofty character.

"While it is in direct opposition to the true history of all human institutions, indeed to the very nature of man, to *always* elegantly and peacefully glide in smooth waters; and, while unpleasant eruptions will occasionally present themselves, let us use our best efforts to make the intervals as great in extent as practicable, to the end that social, professional and scientific harmony may not be seriously disturbed. This great desideratum can only be approached by so amending our laws as to prevent the introduction of such disruptive factors into our general sessions; in fact, all such questions should be removed beyond the jurisdiction of the judicial council, except on appeal from the decisions of the councils of the County and District Associations. A law of this nature would virtually rid our State Association of these unfortunate questions. The medical faculty resident in the counties and districts wherein these troubles materialize, being conversant with all the ins and outs and all the general and special environments, are more competent, it would seem, to honorably and judiciously adjust these claims, than any other parties could be."

